# vSDC: a method to improve early recognition in virtual screening when limited experimental resources are available

**DOI:** 10.1186/s13321-016-0112-z

**Published:** 2016-01-18

**Authors:** Ludovic Chaput, Juan Martinez-Sanz, Eric Quiniou, Pascal Rigolet, Nicolas Saettel, Liliane Mouawad

**Affiliations:** Chemistry, Modelling and Imaging for Biology (CMIB), Centre de Recherche, Institut Curie-PSL Research University, Bâtiment 112, Centre Universitaire, 91405 Orsay Cedex, France; Paris-Sud University, Orsay, France; Inserm, U1196, Orsay, France; CNRS, UMR 9187, Orsay, France; School of Pharmacy, University of Caen, Normandy, Boulevard Becquerel, Caen, 14032 France

**Keywords:** Virtual screening, Standard deviation consensus, vSDC, Gold, Glide, Surflex, FlexX, DUD-E, Calcineurin, Cdk2

## Abstract

**Background:**

In drug design, one may be confronted to the problem of finding hits for targets for which no small inhibiting molecules are known and only low-throughput experiments are available (like ITC or NMR studies), two common difficulties encountered in a typical academic setting. Using a virtual screening strategy like docking can alleviate some of the problems and save a considerable amount of time by selecting only top-ranking molecules, but only if the method is very efficient, i.e. when a good proportion of actives are found in the 1–10 % best ranked molecules.

**Results:**

The use of several programs (in our study, Gold, Surflex, FlexX and Glide were considered) shows a divergence of the results, which presents a difficulty in guiding the experiments. To overcome this divergence and increase the yield of the virtual screening, we created the standard deviation consensus (SDC) and variable SDC (vSDC) methods, consisting of the intersection of molecule sets from several virtual screening programs, based on the standard deviations of their ranking distributions.

**Conclusions:**

SDC allowed us to find hits for two new protein targets by testing only 9 and 11 small molecules from a chemical library of circa 15,000 compounds. Furthermore, vSDC, when applied to the 102 proteins of the DUD-E benchmarking database, succeeded in finding more hits than any of the four isolated programs for 13–60 % of the targets. In addition, when only 10 molecules of each of the 102 chemical libraries were considered, vSDC performed better in the number of hits found, with an improvement of 6–24 % over the 10 best-ranked molecules given by the individual docking programs.

**Graphical abstract:**
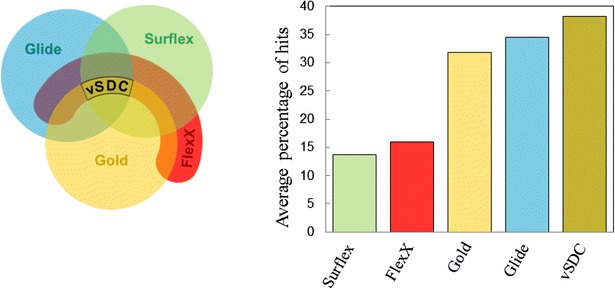
In drug design, for a given target and a given chemical library, the results obtained with different virtual screening programs are divergent. So how to rationally guide the experimental tests, especially when only a few number of experiments can be made? The variable Standard Deviation Consensus (vSDC) method was developed to answer this issue. *Left panel* the vSDC principle consists of intersecting molecule sets, chosen on the basis of the standard deviations of their ranking distributions, obtained from various virtual screening programs. In this study Glide, Gold, FlexX and Surflex were used and tested on the 102 targets of the DUD-E database. *Right panel* Comparison of the average percentage of hits found with vSDC and each of the four programs, when only 10 molecules from each of the 102 chemical libraries of the DUD-E database were considered. On average, vSDC was capable of finding 38 % of the findable hits, against 34 % for Glide, 32 % for Gold, 16 % for FlexX and 14 % for Surflex, showing that with vSDC, it was possible to overcome the unpredictability of the virtual screening results and to improve them

**Electronic supplementary material:**

The online version of this article (doi:10.1186/s13321-016-0112-z) contains supplementary material, which is available to authorized users.

## Background

An increasing number of proteins are identified every year as good therapeutic targets for drug design. This design mainly consists of searching for small molecules that, in most cases, inhibit the protein function, either by inhibiting its enzymatic activity or its protein–protein interaction. When new targets are identified, there are usually no such small molecules known, which requires the use of high throughput screening (HTS) of a chemical library, if the adequate experimental assay exists, to find hits that may constitute good leads for drug design. In many cases, however, such an assay doesn’t exist and the usual strategy is to resort to structure-based virtual screening (VS) when the 3D structure of the protein is known. There is still a caveat, since the docking programs, primarily used in VS, may perform poorly on new targets, if they are different from their training sets. Therefore, a large number of small molecules still have to be tested experimentally. Evaluations of docking programs show that their performance depends on the protein target and the chemical library considered [1–8]. In these evaluation tests, the most commonly used indicators are the receiver operating characteristic (ROC) curve [9], which gives the rate of true positives versus the rate of false positives, and the enrichment factor (EF) [10], which represents the percentage of real active ligands in the top-ranked molecules compared to their percentage in the experimental database. For the ROC curves, the area under the curve (AUC) is the most reported in the publications, giving the overall performance on the entire database [10, 11], whereas for the EF, usually the focus is on the 1–10 % top-ranked molecules [3, 12]. Thus, the aim of the VS evaluations is to find a good proportion of active compounds in 1–10 % of the best ranked molecules, which corresponds to 150–1500 molecules in a medium-sized database of 15,000 compounds.

This usual VS approach can be problematic for the experimentalist interested in a new target. First, for many targets, it may happen that for various reasons, only a small number of in vitro experiments (which may be coupled to in cell experiments) can be carried out to test the activity of the molecules. This is the case of some tests that evaluate the interaction between the target and the small molecules. These tests, like Isothermal Titration Calorimetry (ITC) or NMR studies, are very low throughput experiments, because they are protein and/or time consuming. Therefore, they allow only few molecule tests, reasonably less than 10 for the former and less than 50 for the latter, which is significantly less than the 1–10 % molecules of a medium-sized database as proposed by the evaluation indicators of the VS methods. Second, for a new target, there is little chance to find many inhibitors in an average chemical library since no molecules were developed beforehand for this target. Even with experimental HTS, this chance may be small as observed in several cases [13–15] and as it will be shown below for the cyclin dependent kinase 2 (Cdk2), where the active compounds constitute only 0.5 % of the chemical library.

In this article, our goal is to meet the experimental limitations by finding hits in the 10–50 top-ranked molecules, when the total number of active compounds doesn’t exceed 0.5 % of the database. For this purpose we propose two slightly different consensus VS methods based on standard deviations, the standard deviation consensus method (SDC) and the variable standard deviation consensus method (vSDC), which increase the chance of finding hits. The work was first based on the real cases of two anti-cancer targets, calcineurin (Cn) [16] and a histone binding protein (Hbp). Then the methodology was generalized to a test protein, Cdk2, and to the 102 protein targets of a benchmarking database, the database of useful decoys-enhanced (DUD-E) [17, 18].

Calcineurin is a Ser/Thr phosphatase enzyme that activates the transcription factor NFATc by dephosphorylating it, using the Fe^3+^ and Zn^2+^ ions that are chelated to the active site and their three bound water molecules [19]. Only indirect inhibitors that do not interact with the enzymatic site, like cyclosporine [20] and tacrolimus [21], are known. Our aim was to find new inhibitors that bind specifically to the active site. As for the histone binding protein (which will not be named explicitly for confidentiality reasons), it is involved in DNA replication and has a rather flat and large binding site, with a solvent accessible area of 1868 Å^2^, compared to the surface of the entire protein, 8306 Å^2^. The purpose was the inhibition of its interaction with histones, for which there were no small molecules known. To achieve these two goals, we used VS methods applied to the Institut Curie chemical library (ICCL), which contains about 15,000 virtual compounds, comprising over 8000 real compounds (for the distribution of the physical–chemistry properties of the compounds see http://tiny.cc/o1fu5x).

Cdk2 is a protein kinase for which an experimental HTS was performed using the 8560 real compounds available in the ICCL, yielding 35 active molecules. In this protein, the targeted site was not the usual ATP binding site, but a new allosteric one, located in the C-terminal domain and identified with fluorescence experiments (May C. Morris, personal communication, manuscript in preparation).

The DUD-E [18] is an enhanced and recent version of the popular DUD [17] database that was developed for the benchmarking of VS methods. While DUD only contained 40 protein targets, the DUD-E was enriched to 102 proteins with their 102 chemical libraries. DUD-E was used to evaluate the performance of SDC and vSDC methods.

We will first present the results obtained for Cn and Hbp with the classical VS methods, by applying four commonly-used docking programs [22]. Then, we will explain the SDC method and show its results on the 105 targets cited above. Finally, we will present the related vSDC method and show its results when applied to the DUD-E targets before analyzing its performance with the issue of low-throughput and low-yield experiments.

## Results

### Virtual screening using four docking programs: the results diverge mainly due to the scoring functions

The preparation of the 14,307 molecules of the ICCL was undertaken to find all their enantiomers (when not specified experimentally), protonation states at pH 7.4 ± 1 and tautomers. The resulting 24,186-molecule database was docked in both Cn and Hbp, using the four programs: Glide [23, 24], Surflex [25], FlexX [26] and Gold [27]. Apart from their popularity, the interest of these programs is their variability. Indeed, there are important differences in their search algorithms and their scoring functions (see “Methods”). For each program, when available, both rigid and flexible dockings were carried out and several scoring functions were tested. It should be specified that in all four programs, the small molecule is always flexible and that the qualification of flexible or rigid only concerns the side chains of the protein binding site. For a given program, a high correlation was observed (Additional file 1: Figure S1) between the results of the rigid and flexible dockings (absolute value of the correlation coefficients $\left| r \right| > 0.82$), and between the results of the various scoring functions of a given docking (with $\left| r \right| > 0.92$). Therefore, for simplification, the comparison between the various programs was solely based on the rigid docking results, with one scoring function, Goldscore for Gold, G-score for Glide, and the eponymous Surflex and FlexX.

**Fig. 1:**
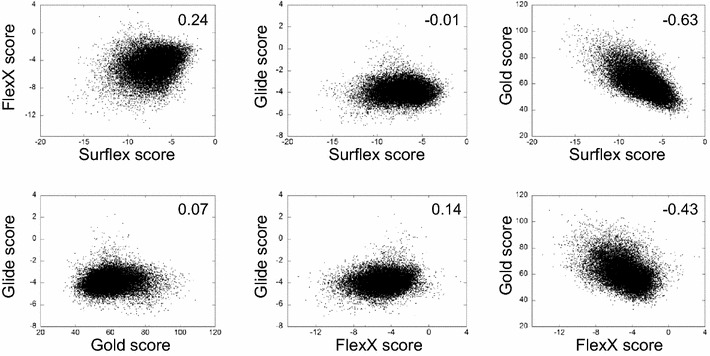
Correlation between the VS results of the four docking programs for Cn. For each compound, the score obtained by one program is reported versus the score obtained with the other program. For Glide, FlexX and Surflex, the scores are given as Δ*G* in kcal/mol, the lowest being the best, whereas for Gold, the score is a positive fitness, and therefore, the highest is the best. The correlation coefficients, *r*, between the programs are given in each plot

The results of the four programs were compared two by two by plotting, for each molecule, its score given by a program versus its score given by the other program (Fig. 1). For Cn, the results were divergent since the correlation observed between the scores given by any two programs was small, except for Surflex and Gold ($\left| r \right| = 0.63$) and FlexX and Gold ($\left| r \right| = 0.43$). However, these fortuitous “good” correlations are mainly dominated by the worst-ranked molecules. Indeed, if only the 50 % best-ranked molecules were considered, the Surflex-Gold and FlexX-Gold correlations would drop to 0.35 and 0.25, respectively. In addition, the correlation between Surflex and Gold is largely due to the correlation of their scoring functions with the number of atoms in the ICCL molecules. Indeed, the absolute value of the correlation coefficient between the scores of these two programs and the number of atoms in the molecules was 0.68 for Surflex and 0.65 for Gold.

For Hbp (Additional file 1: Figure S2), the divergence between the results is accentuated compared to Cn, with smaller correlation coefficients, ranging from 0.02 to 0.26, except for Surflex and Gold, where $\left| r \right| = 0.71$. The latter coefficient, which drops to 0.5 for the 50 % best-ranked molecules, is higher than that of Cn, probably because the correlations between the ranking of the results given by these two programs with the number of atoms in the molecules are also higher than those of Cn, with $\left| r \right|$ equal 0.84 for Surflex and 0.78 for Gold.

A divergence of the scores for the inactive molecules is inconsequential; however, for the top-ranked ones, which are expected to be active, a good correlation is crucial in guiding the experimental tests. Yet, for both Cn and Hbp, the correlations of the top-ranked molecules are too low to be exploitable in proposing molecules for experimental tests. A typical example illustrates well this difficulty: in the case of Cn, the molecule ranked in the first position by Gold, was ranked in the 14,237th position by FlexX, in the 14,290th position by Surflex and not retained at all by Glide, i.e., the molecule that was given as the most active by one program was given as one of the worst molecules by the other three programs. This divergence may be due to either or both the scoring function or the positioning algorithm. To check these assumptions, all the 10 poses given by each program were rescored by the other three programs, i.e., the 967,440 total poses obtained by the four programs were scored and ranked by each of the programs. The divergence in the ranking was maintained (Additional file 1: Table S1) showing that the scoring function was responsible for the divergence of the results, without excluding the role of the positioning algorithms (see the “Discussion” section).

### Visual observation: an unfavorable subjective method

Because of this observed divergence, it is impossible to decide which molecules are to be tested experimentally. Thus, the 1 % top-ranked molecules given by each program, which corresponded to 143 different molecules per program, were visually scrutinized. This approach suggested that the poses given by FlexX were the most plausible ones, based on the number of hydrogen bonds and hydrophobic contacts established between the protein and the molecules. However, most of these molecules were poorly ranked by the other programs, whether this ranking was based on the poses given by their own docking algorithms or after rescoring the FlexX poses (although in the latter case the ranking may be slightly improved, see the “Discussion” section). Thus, it was obvious that this subjective method couldn’t be the way to proceed especially that, for the histone binding protein for instance, the available test was the NMR-HSQC (Heteronuclear Single Quantum Coherence) experiment, in which the reasonable number of tested molecules couldn’t exceed 50. Therefore, from this set of 1 % top-ranked molecules, we only considered the molecules that were common to the four programs. For Cn there were 3 common molecules of which one turned out to be active after the experimental test and for Hbp there were 2 molecules that were inactive. In fact, the cutoff of 1 % was arbitrarily chosen, based on the usual EF analyses, and it should be rationalized in a more objective way.

### The standard deviation consensus method (SDC): finding hits

**Fig. 2:**
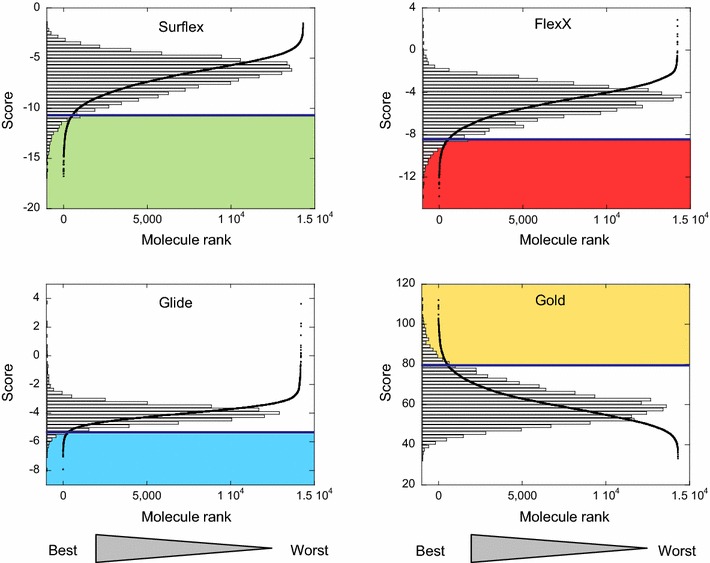
The rank curves of the VS results obtained by the four programs for Cn. In these *curves*, the score obtained for each compound is reported versus its rank given by the same program. The *histogram* of the score distribution is superposed to the rank curve. The *horizontal line* indicates the cutoff of 2× SD that delimits between the stand-out top-ranked molecules (in the *colored boxes*) and the others

**Fig. 3:**
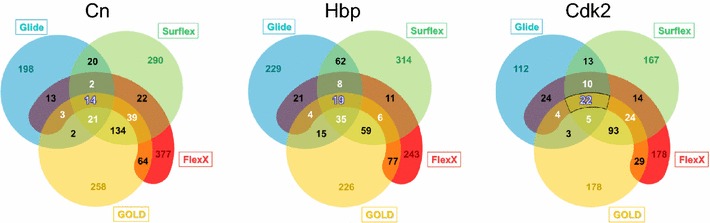
The Venn diagrams for three proteins, Cn, Hbp and Cdk2. For each protein, the diagram shows the number of compounds found within the cutoff of 2× SD, that are common to two, three or four programs, in the intersecting sections. The numbers of the rest of the compounds within this cutoff are reported in the non-intersecting sections. Surflex is in *green*, Gold in *yellow*, FlexX in *red* and Glide in *blue*. As observed, for each protein, the total number of compounds obtained with SDC is different for each program

In order to rationalize the cutoff, the results were plotted as rank curves by reporting the score of the molecules versus their rank (Fig. 2). It can be observed in these curves that the score of some compounds stood out significantly; these compounds are theoretically designated by the programs to be the most active ones. The histograms corresponding to the results were superposed to the rank curves. The best scores that stood out appear to be over two standard deviations (SD) from the average of the curves, as can be observed in Fig. 2. Therefore, based on this value of 2× SD as a new cutoff, we only considered the stand-out molecules in each of the four programs. Their number was variable according to the program. The distribution of these molecules was reported in a Venn diagram (Fig. 3), in which the sets of molecules and their intersections were drawn. The intersection of the sets corresponds to the molecules (beyond 2× SD) shared by several programs. The molecules that were common to the four programs were named consensus molecules (CM) and this approach was called Standard Deviation Consensus (SDC) method. The rationale of this method is that one program may generate errors concerning some compounds, but since the methodologies followed by different programs are different, the chances that they generate the same error is small. Therefore, if we consider the intersection between the sets, we diminish the probabilities of considering erroneous compounds.

For Cn, there were 14 CM, 9 of which were available and tested experimentally by measuring the inhibition of the enzymatic reaction by colorimetry. Three of these molecules were active; they inhibited 50 % of the enzyme activity with a ligand concentration of 20 µM for ligands #1 and #3 and 5 µM for ligand #2 and 40 Units of the protein. Their ranking in the four programs are shown in Table 1. If the nine top-ranked molecules of each program were to be tested experimentally, only two active molecules would have been found by Surflex (the third active molecule was ranked too far), while with the other programs no active molecules would have been found.

**Table 1:** Rank of the active molecules common to the four programs

Ligand	Glide	Surflex	FlexX	Gold
Cn (ligand 1)	116	1	47	206
Cn (ligand 2)	10	3	68	77
Cn (ligand 3)	13	167	453	197
Hbp (ligand 1)	14	265	3	368
Cdk2 (ligand 1)	31	2	5	6
Cdk2 (ligand 2)	61	97	22	7

In the case of Hbp, the rank curves had similar forms as those of Cn (Additional file 1: Figure S3). The SDC method with a cutoff of 2× SD was also applied and the results reported in the Venn diagram (Fig. 3). There were 19 CM, of which only 11 were available for the experimental test. Of these 11 molecules one hit was found; its affinity constant measured by ITC was 1 µM. This hit was ranked in the 14th position by Glide, in the 265th position by Surflex, the 3rd position by FlexX and the 368th position by Gold (see Table 1). This means that for 3 programs, Gold, Surflex and Glide, if only the 11 top-ranked molecules were to be tested experimentally, this hit wouldn’t have been found.

For these two proteins we carried out blind tests because we didn’t have any prior knowledge of the activity of the molecules. However, to better understand how SDC worked, we applied it to Cdk2, a protein for which the experimental results of a HTS were known.

### SDC applied to Cdk2: a good compromise

**Fig. 4:**
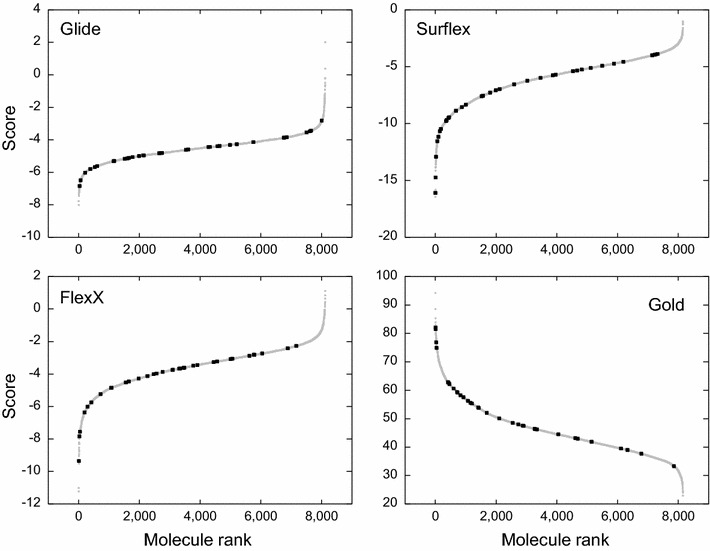
The rank curves of the VS results obtained by the four programs for Cdk2. All the compounds are presented as *grey dots* and the active compounds as *black dots*. As observed, the actives are randomly distributed along the *curves*, except in the region of the worst-ranked compounds, beyond the 8000th rank

For Cdk2 the HTS experiments were carried out on the ICCL and the active molecules were known. There were 35 actives out of 8560 molecules tested. We performed the VS calculation using the same procedure as for Cn and Hbp, but on a reduced chemical library, where only the molecules that were tested experimentally were considered. The number of these molecules after filtering was 8152 (see “Methods”), for which we considered as before, the enantiomers, all protonation states at pH 7.4 ± 1 and tautomers. The binding site that was considered for the VS was not the ATP site, but the allosteric site identified experimentally. The analysis of the VS results allowed us to better understand the distribution of the active molecules. The rank curves are plotted in Fig. 4, in which the active molecules are reported as black dots. It may be observed that in all four programs, the active molecules are distributed almost randomly along the rank curves, except in the range of the very worst molecules. Therefore, the docking enrichment curves (see Eq. 3 below for the definition, Additional file 1: Figure S4) show that they are slightly better distributed than random. The best-scored active molecule is the same in the four programs, although it doesn’t occupy the same rank as can be seen in the penultimate line in Table 1 (Cdk2, ligand 1). When applied, the SDC method with a cutoff equal to 2× SD yielded 22 consensus molecules, of which two were active.

If we were in the situation of a blind test according to which the HTS wasn’t done beforehand and the active molecules weren’t previously known, and if in addition we couldn’t test more than 22 molecules, without the SDC method the degree of success would have depended on the program used. With Glide, there would have been no molecule found (the first hit was ranked 31), with FlexX and Gold, two molecules would have been identified (ranked 5 and 22 for the former, and 6 and 7 for the latter), the same ones as those detected with SDC, whereas with Surflex, 3 molecules would have been detected (ranked 2, 4 and 18). However, these results can’t foretell on the performance of a program because this performance depends on the target. Indeed, whereas Surflex yielded the best results for Cdk2 with 3 hits found in 22 molecules, it ranked poorly the only hit identified for Hbp, and on the contrary, Glide, with which no hit was identified for Cdk2, ranked rather well the hits of Cn (Table 1).

For the three preceding targets, SDC seemed to be a good compromise to obtain at least one hit when few molecules could be tested experimentally, although it gave a less good result than that of Surflex for Cdk2. To monitor the generality of the good performance of SDC we applied it to the 102 protein targets of the DUD-E.

### Application to the DUD-E: SDC performs better than each isolated program

DUD-E [18] is a database of 102 protein targets with a “chemical library” for each protein, composed of active molecules (an average of 224 per protein) and decoys designed for the benchmarking of docking programs. In this database, the average ratio of active molecules to decoys is 1–50, corresponding to 2 % of ligands in each chemical library, which represents a relatively high density of actives. This density may not be realistic in the case of a new protein target, as for instance Cdk2 with its newly identified allosteric binding site, for which the ratio of active/inactive molecules found in the ICCL was less than 1/200, corresponding to about 0.5 %. Since for a new protein target it is more likely to observe this low density of actives than that of the DUD-E, and in order to simulate such a case with the DUD-E, we lowered the ligand-to-decoys density for each target by eliminating randomly several active molecules until the density of 0.5 % was reached. All the following calculations were carried out on this “diluted” database.

**Fig. 5:**
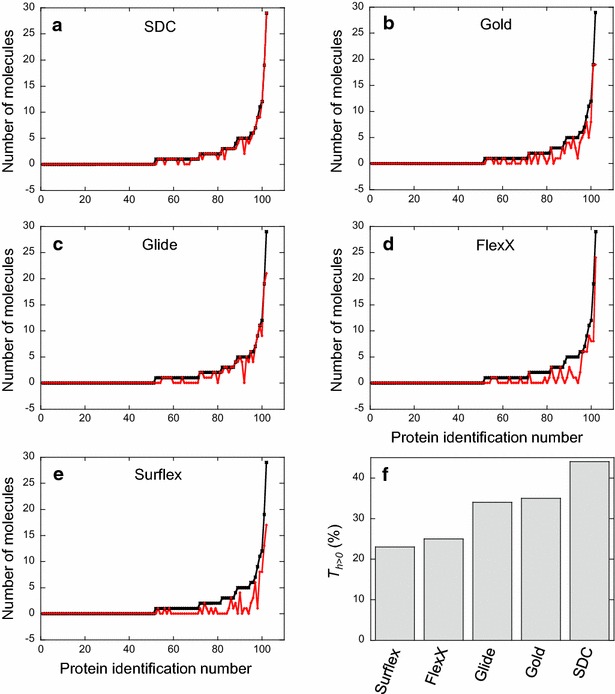
Comparison between the results of SDC and the isolated programs for the DUD-E proteins. **a**–**e** The *black curve* is the same in *all five panels*. *Each black dot* of this *curve* corresponds to the number of molecules (nCM) common to the four programs, within the cutoff value of 2× SD, versus the protein identification number. This identification number was attributed to the proteins to have the *curve* sorted in the ascending order. For each protein, the number of actives found within the nCM (or *n*_*test*_) molecules is represented as a *red dot*. The *red curves* drawn for SDC and the four isolated programs show that there are more actives obtained with SDC, since its *red curve* is the closest to the *black one* (the number of actives is almost equal to nCM). **f** This *panel* summarizes the results observed in *panels* **a**–**e**, by showing for each method the percentage of proteins for which at least one hit was found ($T_{h > 0}$)

The same procedure as described above was applied to the 102 proteins of the DUD-E, by using the programs Glide, Surflex, FlexX and Gold. The SDC method was applied with a cutoff 2× SD. For each protein target, the number of consensus molecules (nCM)—corresponding to the number (*n*_*test*_) of molecules to be tested experimentally in a real situation—was different, ranging from 0 to 29, depending on the target. Furthermore, most of them were real hits, as shown in Fig. 5a, meaning that the consensus molecules were mainly active ones. To compare the performance of SDC with that of each isolated program, as we did for Cdk2, for each protein target we considered the same number (*n*_*test*_) of top-ranked molecules as nCM and counted the number of actives among them. For instance, for the dihydroorotate dehydrogenase (Pyrd in the DUD-E nomenclature), the number of consensus molecules was equal to 5 and they all happened to be active; we then considered the 5 top-ranked molecules of each program and counted the number of hits among them. We found 4 hits with Glide, 3 with Gold, 2 with FlexX and none with Surflex.

In Fig. 5b–e we reported the number of hits found for each target in the *n*_*test*_ best-ranked molecules from each docking program. We observed that each program taken individually performed with less efficiency than the SDC method, since in the same number of molecules (nCM = *n*_*test*_) there was less chance to find hits with any of the 4 programs than with the SDC method. In addition, the performance of the programs was not similar: Glide and Gold seemed to perform better than Surflex and FlexX. However, these results were not satisfactory, since for 50 % of the targets, the number of consensus molecules, thus the number of molecules to be tested experimentally, was equal to zero and consequently, no actives could be found. Because of this lack of consensus molecules, the percentage of targets with hits ($T_{h > 0}$), which is defined as the percentage of protein targets (*T*) for which at least one hit is found ($h > 0$; *h* being the number of hits; see Eq. 1 in the next section), was relatively low (Fig. 5f); it was 44 % for SDC, 35 % for Gold, 34 % for Glide, 25 % for FlexX and 23 % for Surflex, hence the necessity of modifying the procedure.

### Going further: the variable SD consensus (vSDC) method and its application to the DUD-E

In the previously described SDC method, the cutoff value ($c = x \times SD$, where $x = 2$) was chosen by the user resulting in a variable nCM. Since no hits were found in more than half of the cases, the procedure was modified and the strategy reversed. Instead, the number of consensus molecules nCM (which is equivalent to *n*_*test*_) is decided by the user, and the SD multiplier *x* is varied to achieve the expected nCM. The cutoff value is consequently different for each target considered. This method, called the variable standard deviation consensus method, vSDC, was applied to the DUD-E database based on the results of the 4 docking programs.

The classic performance indicators of a screening method, the Area Under the Curve (AUC) of the Receiver Operator Characteristic (ROC) curve [9] or the Boltzmann-Enhanced Discrimination of Receiver Operating Characteristic (BEDROC) [11], especially developed for the early recognition problem, use the entire chemical library for their calculation. Since we are only considering a small number of molecules (*n*_*test*_ ≤ 50), other metrics have to be used.

First, the analysis of the previously defined percentage of targets with hits ($T_{h > 0}$) was considered for a given number of tested molecules, *n*_*test*_.

1 $$T_{h > 0} = \frac{{n_{p}^{h > 0} \, }}{{N_{p} }}$$

where $n_{p}^{h > 0}$ is the number of proteins for which at least one hit was found, and $N_{p}$ is the total number of protein targets. For the DUD-E, $N_{p} = 102$.

Second, to quantify the successful hit identification, the yield of actives (*Y*) and the docking enrichment (*E*) are most commonly used. For a given target, these indicators quantify the probability of finding one active in the *n*_*test*_ selected compounds or in all the actives (*A*) of the database.

2 $$Y = \frac{h}{{n_{test} }}$$

3 $$E = \frac{h}{A}$$

*h* is the number of hits found. However, when only a small fraction of the molecules are tested (*n*_*test*_ ≪ *A*) and all are hits $\left( {h = n_{test} } \right)$, then $Y = 1$ whereas *E* ≪ 1. In this case, the docking enrichment does not reflect the good performance of the method. On the contrary, when the number of molecules tested exceeds the total number of actives ($n_{test} > A$) and all the hits were retrieved (*h* = *A*), then *E* = 1 while *Y* < 1. In this case, it is the yield of actives that does not reflect the good performance of the method. Therefore we propose a corrected yield of actives, which combines the two indicators by replacing the denominator by the lowest of the number of actives and the number of molecules tested, Eq. 4. This denominator represents the maximum number of *findable* hits.

4 $$Y_{C} = \, \frac{h}{{{ \hbox{min} }\left( {A{ ; }n_{test} } \right)}}$$

With this definition of the yield and because the proportion of active-to-inactive molecules is the same in all the DUD-E diluted chemical databases used in this study, *Y*_*C*_ is comparable across the proteins in the DUD-E dataset. Therefore, *Y*_*C*_ was averaged over all the 102 protein targets and the mean value, $\left\langle {Y_{C} } \right\rangle$, for a given *n*_*test*_ molecules, is used to compare the relative efficacy of the methods.

Third, for any protein target of the DUD-E, for a given *n*_*test*_ value, vSDC may find more, or less, hits than each of the considered programs. To obtain a direct comparison between vSDC and the isolated programs, the net balance between the number of protein targets for which vSDC finds more hits than a given program $\left( {n_{p}^{{{\text{vSDC}} > {\text{program}}}} } \right)$ and the number of targets for which the program finds more hits than vSDC $\left( {n_{p}^{\text{program > vSDC}} } \right)$ was calculated. This net balance is reduced to a percentage, Δ*p*, by taking into account the total number of targets, *N*_*p*_.

5 $$\Delta p = \frac{{n_{p}^{{{\text{vSDC}} > {\text{program}}}} {-}n_{p}^{{{\text{program}} > {\text{vSDC}}}} }}{{N_{p} }}$$

When, for a target, vSDC and the considered program perform equally, i.e., they find the same number of hits, this target was not taken into account.

**Fig. 6:**
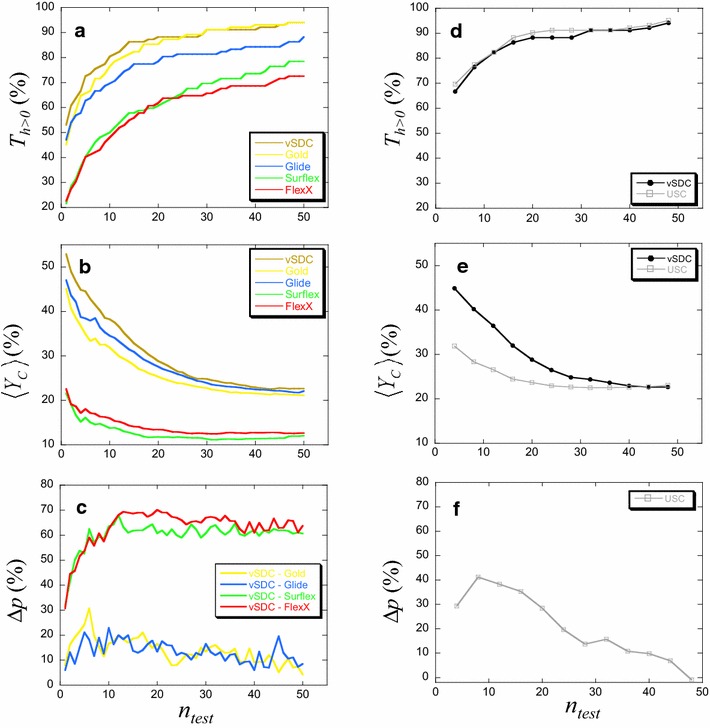
Comparison between vSDC and the isolated programs (**a**–**c**) or USC (**d**–**f**) for the DUD-E proteins. The comparison is based on the three criteria: **a**, **d** the percentage of proteins for which at least one hit was found ($T_{h > 0}$, Eq. 1), (**b**, **e**) the average corrected yield of actives ($\left\langle {Y_{C} } \right\rangle$, Eq. 4) and (**c**, **f**) the percentage of the net balance of proteins (Δ*p*, Eq. 5) for which vSDC is compared to the isolated programs (**c**) or USC (**f**). For the latter criterion, if Δ*p* > 0, the result is in favor of vSDC, otherwise (i.e., Δ*p* < 0) it is in favor of the program

These three criteria, $T_{h > 0}$, $\left\langle {Y_{C} } \right\rangle$ and Δ*p*, were calculated for all *n*_*test*_ values ranging from 1 to 50 and presented in Fig. 6.

As observed in Fig. 6a, the percentage of targets with hits, $T_{h > 0}$, was higher with vSDC than with the isolated programs, Glide, Surflex and FlexX, for all values of *n*_*test*_ under 50. Considering Gold, for *n*_*test*_ ≤ 20, the difference with vSDC was small but meaningful, as it ranged between 2 and 8 % in favor of vSDC. However, for *n*_*test*_ over 20, a difference of ±1 % makes the two methods equivalently performing from this regard. With only 10 molecules to be tested experimentally (*n*_*test*_ = 10), at least one hit was found for more than 80 % of the proteins with vSDC, 76 % of the proteins with Gold, 70 % with Glide, 50 % with Surflex and 48 % with FlexX. For the maximum value of *n*_*test*_ considered (*n*_*test*_ = 50), 94 % of the proteins had at least one hit with vSDC and Gold, 88 % with Glide, 78 % with Surflex and 72 % with FlexX.

On the other hand, the mean value of the corrected yield of actives, $\left\langle {Y_{C} } \right\rangle$, obtained with vSDC was significantly higher than that of the isolated programs, Gold, Surflex and FlexX (Fig. 6b), considering their error intervals, but not Glide (the error bars are not shown in the Figure for the sake of clarity). However, the difference with Glide showed a clear trend in favor of vSDC, because its curve was systematically above that of Glide. For *n*_*test*_ = 10, on average 38 % of the findable hits were found with vSDC, 34 % with Glide, 32 % with Gold, 16 % with FlexX and 14 % with Surflex. For all these cases, the error was about 3 %. For *n*_*test*_ = 50, on average 23, 22, 21, 13 and 12 % of the findable hits were found with vSDC, Glide, Gold, FlexX and Surflex, respectively, with an error of about 1 %.

Finally, considering the percentage of the net balance of proteins, $\Delta p$, Fig. 6c shows that it was in favor of vSDC ($\Delta p > 0$) in all cases. For *n*_*test*_ between 10 and 50, more hits were found with vSDC than with Glide for an average of 13 % of the targets, with Gold for 14 %, with Surflex for 60 % and with FlexX for 65 % of the targets.

### A complementary approach: the union of the four program results (USC)

The vSDC method amounts to considering the *intersection* of subsets of molecules taken from the four programs. We may ask if it would not be more efficient to consider instead the *union* of subsets of molecules. To test this possibility, once the number *n*_*test*_ of molecules chosen, the *n*_*test*_/4 top-ranked molecules were gathered from the four programs. This procedure, which will be called USC, for the United Subset Consensus, necessitates *n*_*test*_ to be a multiple of the number of programs considered (4 in our case) to have an equal number of molecules (*n*_*test*_/4) coming from each program. The results are compared to those of vSDC in Fig. 6d–f. It may be observed that USC gave a percentage of proteins with hits, $T_{h > 0}$, equivalent to that of vSDC (95 % for *n*_*test*_ = 48), whereas the average yield of actives, $\left\langle {Y_{C} } \right\rangle$, is less effective for USC and the percentage of the net balance of proteins, Δ*p*, is clearly in favor of vSDC, although the difference diminishes with increasing *n*_*test*_ until it becomes slightly in favor of USC for *n*_*test*_ = 48. We tested both methods for a number of molecules between 52 and 100 and observed that then USC finds more hits for an increasing number of proteins. For instance, when *n*_*test*_ = 100, for 55 proteins the results of USC are better than vSDC, whereas for 32 proteins vSDC performs better than USC. For the remaining 15 proteins, the same number of hits was found.

This shows that, for a small number of molecules it is more useful to use vSDC, while for *n*_*test*_ over 48, it may become more interesting to consider the union of 4 small sets of top-ranked molecules (*n*_*test*_/4). In both cases, there is still the need of using four VS programs.

### vSDC method used with two programs with homogeneous performance

As can be observed in Fig. 6a–c, the performance of the four programs on the protein targets of the DUD-E was not equivalent. Gold and Glide seemed to perform better than Surflex and FlexX. Therefore, the question was to which extent the poor performance of Surflex and FlexX influenced the results of vSDC. To answer this question we did the test on the targets of the DUD-E by only considering Gold (g) and Glide (g) on the one hand or Surflex (s) and FlexX (f) on the other hand, to find the consensus molecules. The results of vSDC_gg_ and vSDC_sf_ were compared to those of the corresponding isolated programs, using the three criteria mentioned in “Going further: the variable SD consensus (vSDC) method and its application to the DUD-E” section.

**Fig. 7:**
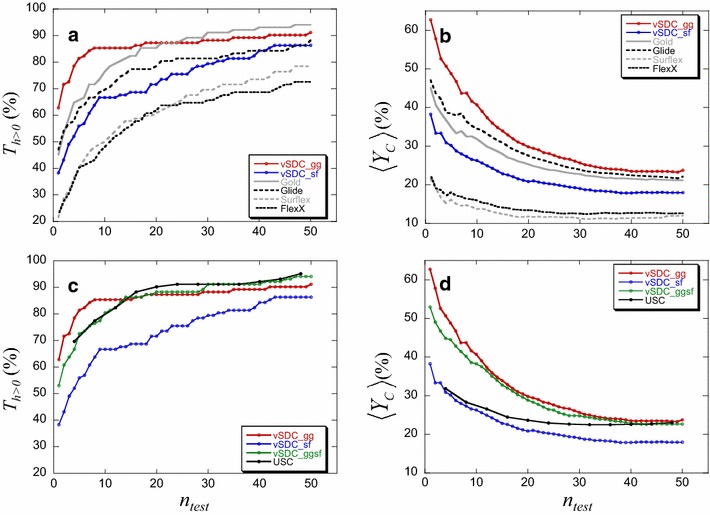
Comparison between vSDC based on two programs and all the other methodologies for $T_{h > 0}$ and $\left\langle {Y_{C} } \right\rangle$. vSDC_gg_ (or vSDC_gg) is based on Gold and Glide (gg) while vSDC_sf_ (or vSDC_sf) is based on Surflex and FlexX (sf). **a**, **b** Comparison with the isolated programs. **c**, **d** Comparison with vSDC_ggsf_ and USC

**Fig. 8:**
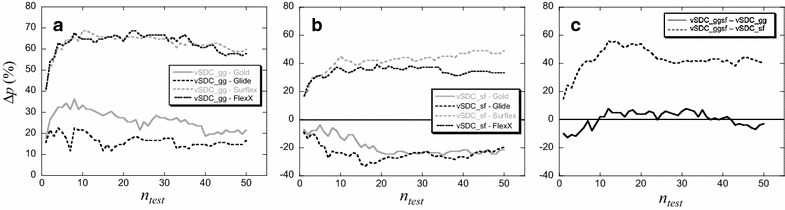
Comparison between vSDC based on two programs and all the other methodologies for Δ*p*. **a**, **b** vSDC_gg_ and vSDC_sf_, respectively, are compared to all the isolated programs, even those that were not used for the consensus. **c** vSDC_ggsf_ is compared to vSDC_gg_ and vSDC_sf_

Considering vSDC_gg_, the percentage of targets with hits, $T_{h > 0}$ (Fig. 7a) was much higher than that of Glide for all *n*_*test*_ ≤ 50, and significantly higher than that of Gold for *n*_*test*_ ≤ 16, with a difference ranging from 3 to 19 %, whereas for *n*_*test*_ between 25 and 50, the curves are reversed, and the difference is between 1 and 4 % in favor of Gold. For the average of the yield of actives, $\left\langle {Y_{C} } \right\rangle$, (Fig. 7b) the curves showed a clear trend in favor of vSDC_gg_, which was significantly higher than Gold and Glide for *n*_*test*_ ≤ 10, considering their error bars (which are not shown for the readability of the curves). For the net balance of proteins, Δ*p*, (Fig. 8a), when *n*_*test*_ is between 10 and 50, more hits were found with vSDC_gg_ than with Glide and Gold for, respectively, an average of 15 and 25 % of the targets. Therefore, vSDC based on only Gold and Glide, improved significantly the VS results from the perspective of the 3 criteria except for the percentage of proteins with hits for *n*_*test*_ over 25.

Considering vSDC_sf_, the improvements of the results compared to those of the isolated programs Surflex and FlexX are still more obvious. Indeed, there are important gaps separating the curves of vSDC_sf_ from those of Surflex and FlexX in Fig. 7 panels a, b. In addition, Δ*p*, presented in Fig. 8, showed that, when *n*_*test*_ is between 10 and 50, more hits were found with vSDC_sf_ than with FlexX and Surflex for, respectively, an average of 35 and 44 % of the targets. For information, we also compared the results of vSDC_sf_ with those of the isolated Gold and Glide, although these two programs were not included in the vSDC_sf_ calculation. Despite the remarkable improvements of the results of Surflex and FlexX brought by vSDC_sf_, the performance of the latter was still below that of Gold and Glide, separately.

The results of vSDC based on two programs, i.e., vSDC_gg_ and vSDC_sf_, were also compared to those of vSDC based on the four programs, i.e., vSDC_ggsf_ (Figs. 7c, d, 8c). All the three criteria were significantly in favor of vSDC_ggsf_ when compared to vSDC_sf_ but they were more questionable when compared to vSDC_gg_. Indeed, vSDC_gg_ > vSDC_ggsf_ for *n*_*test*_ ≤ 15, considering $T_{h > 0}$, and vSDC_gg_ ≥ vSDC_ggsf_ for all *n*_*test*_ ≤ 50, considering $\left\langle {Y_{C} } \right\rangle$. Finally, $\Delta p < 0$ for *n*_*test*_ < 10 and *n*_*test*_ > 40. In this case, the two methodologies may be considered close regarding their performance.

Since the performances of vSDC_ggsf_ and vSDC_gg_ are equivalent, we may wonder if the use of vSDC based on only two programs as Gold and Glide wouldn’t be sufficient. Generally, this may be true but, first, without a prior knowledge of the performance of the programs for the considered target, it is not easy to choose the two best-performing programs (for a few targets, these programs are Surflex and FlexX), and second, there are some cases shown in Table 2 that demonstrate that the use of only Gold-Glide is not always optimal. Indeed, for Ampc and Thb, for instance, the elimination of two less performing programs (in this case Surflex and FlexX) from vSDC degraded the results, since for the former target vSDC_gg_ = 2 hits while vSDC_ggsf_ = 4, and for the latter target vSDC_gg_ = 16 hits while vSDC_ggsf_ = 20. Note that for both Ampc and Thb targets, vSDC_sf_ = vSDC_gg_ although the performance of the isolated Surflex and FlexX programs (with 0 hit found for the former and 7 and 6 hits found, respectively, for the latter) is less efficient than that of Gold and Glide (with 1 hit for Ampc and 15 and 14 hits for Thb). Conversely, when the four programs present equivalent performance, it could be expected that the elimination of two of them from vSDC may degrade the results. However, this is not necessarily the case like for Cp3a4 and Thrb. For Cp3a4, although the performance of Glide (with 1 hit) is slightly lower than that of Gold, Surflex and FlexX (with 2 hits), vSDC_gg_ gives the highest number of hits (3), while vSDC_sf_ = vSDC_ggsf_ = 2; for Thrb, although the number of hits given by each couple of programs is almost equivalent (16 and 28 for Gold and Glide, and 17 and 25 for Surflex and FlexX), vSDC_sf_ = 43, while vSDC_gg_ gives only 22 hits and vSDC_ggsf_ = 33. On the other hand, it is worthy to note that the greatest number of hits obtained for any protein of the DUD-E with an isolated program was given by FlexX for Try1 (37 hits in the 50 best-ranked molecules), despite the fact that the general performance of this program was lower than the three others. This demonstrates the difficulty of choosing one program to use. For this target, based on the results given by Surflex and FlexX, vSDC_sf_ yielded 49 hits for *n*_*test*_ = 50.

**Table 2:** Examples to illustrate particular results with 2 and 4 programs

Protein	Number of hits found in *n*_*test*_ tested molecules							
	Gold	Glide	Surflex	FlexX	vSDC_gg_	vSDC_sf_	vSDC_ggsf_	USC
Ampc (β-lactamase)	1	1	0	0	2	2	4	0
Thb (thyroid hormone receptor β1)	15	14	7	6	16	16	20	10
Cp3a4 (cytochrome P450 3A4)	2	1	2	2	3	2	2	2
Thrb (thrombin)	16	28	17	25	22	43	33	25
Try1 (trypsin I)	26	28	28	37	32	49	45	36

The proteins are named according to their abbreviations given in the DUD-E and their function in brackets. All examples are based on *n*_*test*_ = 50, therefore, for USC the results were the average between *n*_*test*_ = 48 and *n*_*test*_ = 52

## Discussion

In VS studies, the use of only one program can’t be recommended except if this program is known to perform well for active sites similar to the one that is targeted. Without this prior knowledge, there is no rational basis for the choice of the program to use, while this choice is crucial for the quality of the results. Indeed, the ranking of the small molecules is highly dependent on the program, as observed in Fig. 1 and Additional file 1: Figure S2, for Cn and Hbp, respectively, where a divergence of the results is observed. Similar divergence was observed for all the targets, including those of the DUD-E (data not shown). However, for the three targets for which the docking was done on the ICCL, and only for these targets, a correlation coefficient $\left| r \right| \ge 0.6$ was observed between the results of Gold and Surflex. This correlation concerned mainly the bottom-ranked molecules and is probably due to the correlation of the two scoring functions with the number of atoms in the small molecules. Indeed, the correlation coefficient between the ranking results of Gold or Surflex on the one hand and the number of atoms of the molecules on the other hand was greater than 0.65. This correlation was only observed for the docking done on the ICCL which presents a great diversity of molecules (in size, charge, shape …), whereas for each protein target of the DUD-E, the same divergence was observed between Gold and Surflex as between all the other programs, since each corresponding “chemical library” consists of molecules of homogeneous sizes. Therefore, we may consider that the discrepancy between the VS results is general. The divergence may be due to the scoring functions that didn’t rank the molecules in a similar way, to the positioning algorithms that couldn’t find similar poses and consequently didn’t allow similar ranking, or to both the scoring functions and the positioning algorithms. To address this question, the re-scoring procedure was applied to Cn, Hbp and Cdk2 using all the poses (not only the best ones) given by the four programs. The results showed that even when the poses were exactly the same, the ranking of the molecules by the different programs was still divergent due to the scoring functions. However, in spite of this divergence, considering a better pose improved the ranking of some molecules. This was for instance the case of Hbp and its identified hit: when its plausible pose given by FlexX was re-scored by the other programs, the rank of the molecule was improved, going from 14 to 4 with Glide, from 265 to 61 with Surflex and from 368 to 90 with Gold. This shows that both the scoring function and the positioning algorithm are responsible at different levels for the divergence of the results, which raises the question of the reliability of the docking programs and the way to make use of them to find hits for new targets.

The VS results for all the 105 protein targets considered in this study and the 103 chemical libraries (ICCL being used for three targets) showed that the performance of each program depended on the target considered and even on the small molecules docked into this target. Indeed, while for the DUD-E proteins, the performance of Surflex and FlexX was rather limited, these programs performed the best for Cn and Hbp, respectively. On the other hand, for the same target, for instance Cdk2, Surflex was the best in finding the first ligand and the worst for the second ligand (Table 1). Therefore, it is useful to consider some consensus between the programs.

What is usually meant by consensus is the consensus score which combines several scoring functions based on the same positioning of the molecules [28–32], or on the ranking of clusters of molecular poses [33, 34]; however, the improvements due to these methods are disputed in the literature [3, 35–37]. Here, the consensus is made between the ranking results of several VS programs, with their variety of positioning and scoring. For this consensus, we chose to use four programs in order to minimize the probability of obtaining the same false positive molecules in the top-ranked ones, considering that this probability should be reduced with the increasing number of programs, because, with their various positioning algorithms and scoring functions, programs have little chance to commit the same mistakes. To find the consensus molecules there were several ways to decide which cutoff to use. Charifson et al. [38] tested an arbitrary number of 300 top-ranked molecules. With such a cutoff, the third hit of Cn and the only hit of Hbp would have been missed, since these ligands are ranked beyond the 300^th^ position in at least one program. Here we tried the 1 % top-ranked molecules, which was also an arbitrary number, and the result didn’t allow us to find any hit for Hbp and only one hit for Cn. In the SDC method, we chose the cutoff to be equal to 2× SD, based on the shape of the rank curves. As shown in the Venn diagrams (Fig. 3), this method, when applied to only two or three programs, may generate a great number of common molecules, too big for the experimental tests, as for instance in the case of Cn, where there were 208 molecules in common between Surflex and Gold (14 + 39 + 21 + 134) or 53 molecules in common between Surflex, Gold and FlexX (14 + 39). The introduction of the fourth program was useful to diminish the number of molecules of interest, justifying a posteriori the use of four programs, whereas a fifth one didn’t seem necessary, because the number of common molecules, nCM, would be close to zero. Although the SDC method, based on Gold, Glide, Surflex and FlexX, yielded good results for Cn, Hbp and Cdk2, as it allowed us to find hits for these proteins with little experimental tests, it wasn’t effective enough for the DUD-E proteins, where no hits were found for 50 % of the targets (because nCM was then equal to 0), hence the development of vSDC.

In the vSDC method, the cutoff is a variable number of SDs gradually decreased until the defined number of common molecules (nCM = *n*_*test*_) is found. Since this method consists of gathering the *n*_*test*_ molecules that are common to several programs, it somehow amounts to bringing these molecules from their rather far rank (1700 on average) to the first ranks (less than 50). Indeed, to find the common molecules, vSDC has to go very far in the ranking given by the programs; depending on the protein and the degree of divergence of the docking results, this ranking ranges from more than 400 for the “easiest” case to more than 7000, for the “hardest” one. This large exploration allows vSDC to improve the docking results.

With vSDC the chance of finding at least one hit for the DUD-E targets was between 80 and 94 % when testing 10 to 50 molecules (Fig. 6a). Even for *n*_*test*_ ≤ 10, the percentage of targets with at least one hit, $T_{h > 0}$, was still good since it concerned the majority of the proteins and more precisely $T_{h > 0}^{vSDC}$ was comprised between 53 and 80 %, which was higher than the percentage of targets $T_{h > 0}^{Gold}$ obtained with the best performing program according to this criterion, i.e., Gold, where, in the same *n*_*test*_ interval, $T_{h > 0}^{Gold}$ was between 45 and 76 %. However, for $20 < n_{test} \le 50$, $T_{h > 0}^{vSDC}$ and $T_{h > 0}^{Gold}$ were similar, with a maximum value of 94 %. Note that, for all *n*_*test*_ under 50, the percentage of targets with hits obtained with vSDC was significantly higher than that obtained with the other three programs, where the maximum reached was only 88, 78 and 73 % of the targets for Glide, Surflex and FlexX, respectively. These results are summarized in Additional file 1: Figure S5 and may be presented as follows, considering *T*_*h*>*0*_: vSDC ≥ Gold > Glide > Surflex > FlexX.

As observed in Fig. 6, depending on the chosen criterion the performances of Gold and Glide are reversed, as well as those of surflex and FlexX. Indeed, considering the percentage of proteins with at least one hit, Gold > Glide and Surflex > FlexX, but considering the average corrected yield of hits, $\left\langle {Y_{C} } \right\rangle$, Glide > Gold and FlexX > Surflex. However, with this criterion, vSDC does better than the four isolated programs, i.e., $\left\langle {Y_{C} } \right\rangle$: vSDC > Glide > Gold > FlexX > Surflex. The results are also summarized in Additional file 1: Figure S5.

Considering the percentage of the net balance of proteins (Δ*p*), for an average of 13–14 % of the DUD-E proteins there were more hits found with vSDC than with the best performing programs, Glide or Gold (Fig. 6c); Δ*p*: vSDC > Gold ≈ Glide > Surflex ≈ FlexX. The three criteria, $T_{h > 0}$, $\left\langle {Y_{C} } \right\rangle$ and Δ*p*, showed that for any number of consensus molecules between 1 and 50, vSDC based on Gold, Glide, Surflex and FlexX performs better than any of these isolated programs (or similarly, only in the case of Gold, for $T_{h > 0}$ in the range of $n_{test}$ between 20 and 50).

Since in vSDC the user sets the number of molecules to test, the possibility of combining fewer programs was explored. For this purpose we used two homogeneously-performing programs, i.e., Gold–Glide and Surflex–FlexX. In the latter case, since the performance of the two programs was in general rather limited, there was a large room for improvement and the vSDC_sf_ performance was significantly higher than that of the isolated Surflex and FlexX, according to any of the three criteria. Conversely, Gold and Glide performed rather well, and therefore, the room for improvement was narrow. Despite that, the performance of vSDC_gg_ was still better than that of Glide considering $T_{h > 0}$, $\left\langle {Y_{C} } \right\rangle$ and $\Delta p$ for all $n_{test} \le 50$ and of Gold considering $\left\langle {Y_{C} } \right\rangle$ and $\Delta p$ for all $n_{test} \le 50$ and $T_{h > 0}$ for $n_{test} \le 20$. The comparison between vSDC_gg_ and vSDC_ggsf_ shows that, based on the three criteria, there is a net gain in using vSDC_gg_ for $n_{test} \le 15$, but this gain is reversed for $T_{h > 0}$ when $n_{test} > 30$.

We also asked if it would be more effective to use the union of small subsets of molecules taken from each program (USC) rather than the intersection of bigger subsets (vSDC). The results show that, for $T_{h > 0}$ the performance of USC is similar to that of vSDC, whereas for $\left\langle {Y_{C} } \right\rangle$ and $\Delta p$, the performance of vSDC is much better. However, for $n_{test} \ge 48$ (at least until *n*_*test*_ = 60, data not shown), all the curves are reversed in favor of USC, compared to vSDC and the isolated programs, but this is beyond the number of molecules considered as reasonable for several experimental tests.

The program to calculate vSDC is downloadable on http://tiny.cc/gegu5x. It allows the finding of *n*_*test*_ consensus molecules specified by the user, based on 2 to 12 different docking program results.

## Conclusion

Structure-based VS programs yield divergent results. Therefore, the choice of the program or the method is crucial for the success of the drug design rationalization. If a program is known to give satisfactory results for protein binding sites similar to the one that is targeted, it may be used alone, but if no such information is available, the performance of the programs would be unpredictable. In some particular cases, this performance may be the opposite of what is expected from the benchmarking made on protein databases, i.e., the less performing program may become the most efficient in finding ligands and vice versa. In addition, if few experimental tests can be performed for various reasons, it is risky to rely on the results of only one program. Therefore, to put the odds on one’s side it is preferable to use several programs and combine the results like with the vSDC method. Although this method depends on the performance of the programs used, it generally improves significantly the results of each isolated program, as observed above from the statistics of the 103 test-proteins considered (102 from the DUD-E and Cdk2 new site) and from the special cases of the phosphatase and the histone binding protein. Although for very early recognition ($n_{test}$ under 15) vSDC based on only Gold and Glide may yield better results than vSDC based on the four programs, we still prefer the use of vSDC with four programs, because with this method, for all $n_{test}$ under 50, there are significant chances of improving the results. However, when $n_{test}$ exceeds 50, the use of USC may be more advantageous.

## Methods

### Target preparation

The crystal structure of calcineurin, at 2.10 Å resolution, was used (PDB 1AUI) [39], from which only the catalytic chain CnA (residues 14–373) was considered, without the autocatalytic domain (residues 469–486). In this chain the two ions (Fe^3+^ and Zn^2+^) and their three bound water molecules that are important for the dephosphorylation reaction were kept for docking. The crystal structure of Hbp was also taken from the PDB for docking. In this structure there were no crystal water molecules. For Cdk2, the crystal structure (PDB 1FIN) [40] solved at 2.30 Å resolution, in which Thr160 is not phosphorylated, was considered and only the catalytic chain was used for the docking. The DUD-E database [18] that consists of 102 protein targets corresponding to diverse functions (kinases, proteases, nuclear hormone receptors, GPCR, etc.), provided all the crystal structures of the receptors, with their crucial cofactors, ions or crystal waters.

All the above targets were prepared using the “Protein Preparation Wizard” module of the Maestro software [14]. This preparation started by inspecting the protonation state of the binding site residues, then adding the hydrogen atoms and optimizing the side chain orientations. We kept relevant water molecules, metal ions and cofactors located in the binding sites. Then, we exported a mol2 format file for each target which was the same starting structure for the four docking programs.

### Ligand preparation

For Cn, Hbp and Cdk2, we performed virtual screening using the ICCL. In this library we removed all the compounds with molecular weight over 750 g mol^−1^ or consisting of less than 10 atoms. The molecules chelating exotic atoms (Au, Cu, Hg, I, Sn, …) or ions (Fe^2+^, Mg^2+^, …) were also filtered because they were not correctly handled by the docking programs. Finally 856 of the 15,163 compounds were removed and the virtual screening was performed on 14,307 unique compounds. The preparation of the chemical library for docking required different steps to generate accurate 3D molecular structures. When chirality was not specified in the chemical library, all the possible enantiomers were generated. In addition, the protonation states of the compounds were adjusted according to the pH of the medium surrounding the target. In our case, the physiological pH at 7.4 ± 1 was retained. All the protonation states with a probability of existence over 10 % at the given pH were generated as well as all the likely tautomers. The preparation of these compounds was achieved using LigPrep 2.8.0 (Schrödinger). After this preparation, the library amounted to 24,186 structures. Since some docking programs, such as GOLD, do not alter bond lengths and angles, thereby the ligands energy was minimized using the OPLS2005 force field to ensure proper bond distances and angles. We generated one conformer per molecule, the exploration of the ligand conformational space being managed by each of the four docking programs.

The DUD-E database includes the multi-mol2 files of active compounds and decoys for each target, which were prepared by the DUD-E team, with their enantiomers, protomers and tautomers [18]. We used them without any modification.

### Docking

The starting point for defining residues of the binding site was to select all residues with at least one heavy atom within 6 Å from the ligand present in the crystal structure of the target. Then, we added every residue beyond 6 Å that we considered as essential to delimit the binding cavity based on a careful visual inspection. We used this residue selection to specify the binding site in FlexX, Gold and Surflex, and to center the required box for docking with Glide. For Cn, Hbp and Cdk2, both the rigid and flexible dockings were used with Gold and Surflex. These four programs use various positioning algorithms and scoring functions. The search algorithm of Glide is a multistep procedure based on a grid positioning followed by MC refinement; for Surflex, it is a Fragment/Surface based algorithm guided by a protomol, which is a negative fingerprint of the protein binding site; for FlexX, it is a fragment based algorithm; and for Gold, it is a genetic algorithm. The scoring functions of the first three programs are various empirical potentials, whereas that of Gold is a force-field-like potential. For more details see references [23] for Glide, [41] for Surflex, [26] for FlexX and [21, 42] for Gold.

These four docking programs proceed to a systematic ligand conformational search, so, the screened compounds are always flexible. With Surflex and Gold, it is also possible to explore the flexibility of the target. For Cn, Hbp and Cdk2, docking with both the rigid and flexible target were used with Gold and Surflex.

The ranking of the molecules obtained with both procedures being similar, only the rigid docking was adopted for the rest of the study, especially as the flexible docking was too much time consuming. In this rigid docking, the structure of the target was held fixed; the side chains were not allowed to rotate. Ten poses per ligand were saved from each program. The molecules were ranked by score.

#### Glide version 6.3

In the case of Glide, ligands are docked in two user-predefined boxes, one inside the other. The inner box represents the space in which the center of each ligand is going to be placed and the outer box defines the limits that the whole reconstituted ligand can occupy. For each target, the center of the boxes and their dimensions were defined manually in order to include in the outer box all the side chains of the active site as defined above.

As recommended in the manual, we used Glide with the standard precision (SP) docking mode. The Epik “state penalty” was not used for the ligands of the DUD-E, because the results with and without this penalty strongly correlate as observed for Hbp (Additional file 1: Figure S1), and therefore we considered that its benefit was too small for a time-consuming procedure. The docking scores are given as ΔG (kcal/mol).

#### Surflex version 2.745

In the preparation of the binding site, Surflex uses an idealized representation of a ligand, called “protomol”, which includes all possible interactions with the binding site such as hydrophobic contacts and hydrogen bonds, via molecular probes (CH_4_, NH and CO). The protomol generation requires two parameters: the “threshold”, which was set to the default value 0.5, and the “bloat” which depended on the binding site. For Cn and Hbp it was equal to 4 Å, for Cdk2 it was equal to 6 Å, and for the DUD-E targets, two separate dockings were performed, one using a bloat value of 6 Å and the other with a bloat of 2 Å. The results were mainly similar, with a slightly better performance with 2 Å for some proteins. Therefore, for the results, only the latter case was considered. The docking mode GeomX, which turns on more exhaustive docking accuracy parameter set, was employed (see Surflex manual on http://www.biopharmics.com). The docking scores given as pK_d_ were converted in ΔG (kcal/mol).

For the flexible docking, and based on the rigid docking results, Surflex offers the opportunity to slightly minimize the potential energy of the side chains of the protein binding site in the presence of the docked compounds, in order to refine and rescore the docking pose.

#### FlexX version 2.1.5

We used the program FlexX provided with LeadiT (BioSolveIT). The selection of the base fragments was set to automatic mode and the fragment placement used the standard algorithm (option 3). We performed a local optimization of 1000 steps with an expanded radius of 3 Å for all poses. The docking scores are given as ΔG in kJ/mol and converted to kcal/mol.

#### GOLD version 5.1

The genetic algorithm parameters were set to auto mode. We performed the docking with the ChemPLP [43] scoring function, which gives the highest success rates for the ligand positioning. Then the poses were scored with both ChemPLP and Goldscore functions. For Cn, Hbp and Cdk2 the results of both functions were correlated (Additional file 1: Figure S1). For the targets of the DUD-E, Goldscore yielded more successful results (data not shown), therefore, only Goldscore was retained for the analyses. The scores are given as fitness positive numbers.

The target flexibility feature proposed by Gold is different from that of Surflex. It consists of pre-defining a library of side chains rotamers for a set of key residues in the binding site which will be explored during the docking process.

### Rescoring procedure

For Cn and Hbp, the total of 967,440 poses obtained by the four programs were rescored and re-ranked by each of the programs, using the same parameters as for docking. During this rescoring procedure, Gold, Glide, Surflex and FlexX allow the refinement of the docking poses, i.e., a slight modification of the location or conformation of the small molecules (the protein is kept rigid). We used this function in order to allow the optimization of the scoring functions and therefore to avoid the bad scores of poses solely due to the difference of the nature of the scoring functions.

### Data analysis

Ranking of the compounds obtained by the different programs were compared by calculating the Spearman’s rank correlation coefficient, using the program R [44] (http://www.r-project.org). The Spearman’s equation appeared more suitable than the Pearson correlation coefficient because we focus on the ranking rather than on a linear correlation between the scores. Indeed, the different scoring functions output are given in heterogeneous units such as binding affinity energy or arbitrary units. In the case of this study, Pearson and Spearman’s correlation coefficient calculations gave similar values (data not shown).

### SDC, vSDC and USC methods

After performing the docking with the four programs, the average and standard deviation (SD) of the scores given by each program are calculated. For the consensus, the cutoff is chosen to be equal to $x \times {\text{SD}}$. In SDC, *x* is fixed (here $x = 2$) and therefore, nCM is variable, whereas in vSDC, nCM is fixed, and therefore, *x* is variable. In the latter case, to obtain the required number of consensus molecules, an iterative search is performed, starting from $x = 3.5$ and decreasing *x* by 0.001 until the fixed nCM = *n*_*test*_ is reached.

For USC, the union of molecule sets from the four programs was considered. For this purpose, *n*_*test*_/4 molecules were taken from each program. When there were molecules in common to several programs, additional molecules were picked in order to get the closest to the desired *n*_*test*_.

## Additional file


10.1186/s13321-016-0112-z The additional file contains all the supplementary material, i.e. five Figures and one Table.

## Abbreviations

SD: standard deviation
SDC: standard deviation consensus
vSDC: variable standard deviation consensus
USC: United Subset Consensus
CM; nCM: consens molecules; number of CM
VS: virtual screening
DUD-E: database of useful decoys-enhanced
EF: enrichment factor
ROC: receiver operating characteristic
AUC: area under the curve
ICCL: Institut Curie Chemical Library
Cn: calcineurin
Hbp: histone binding protein
Cdk2: cyclin-dependant protein kinase 2
HTS: high-throughput screening
ITC: isothermal titration calorimetry
NMR: nuclear magnetic resonance
