# Supplementary material for: vSDC: a method to improve early recognition in virtual screening when limited experimental resources are available
Source: J Cheminform. 2016 Jan 18;8:1. doi: 10.1186/s13321-016-0112-z (PMC4722699; doi:10.1186/s13321-016-0112-z)
Supplement: Supplementary file 1 — 10.1186/s13321-016-0112-z The additional file contains all the supplementary material, i.e. five Figures and one Table. [file 13321_2016_112_MOESM1_ESM.docx]

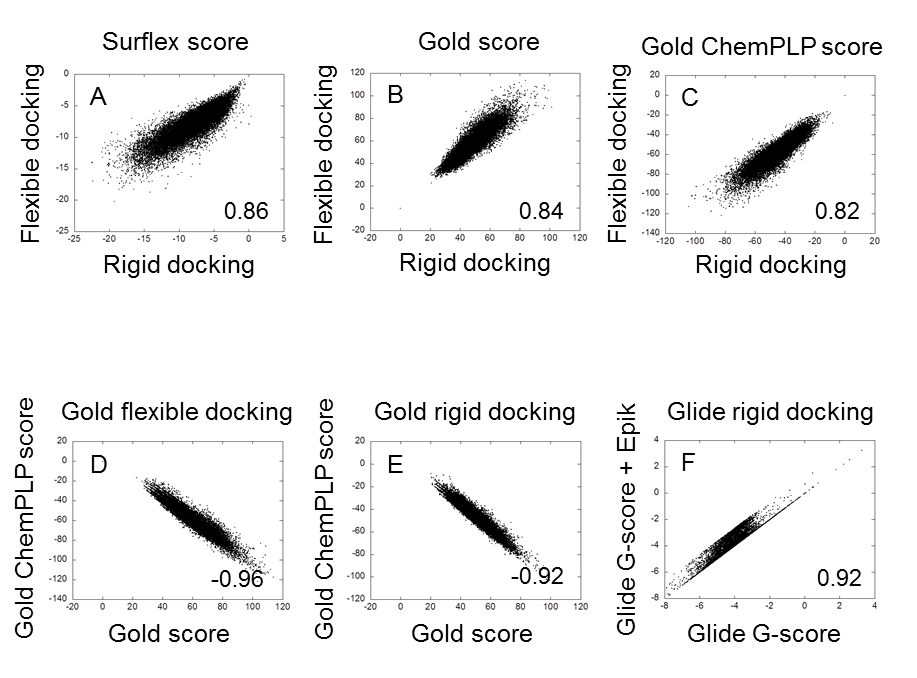


**Figure S1.** Correlation between the VS results for Cn. (**Upper panels**) Correlation between the flexible and rigid dockings performed with Surflex (**A**), Gold using Goldscore (**B**) and Gold using ChemPLP score (**C**). This important correlation is not surprising for Surflex, considering that with this program, the flexible docking consists of slight readjustments of the protein side chains, whereas it is more surprising for Gold, where the side chains are allowed to rotate during the docking. (**Lower panels**) Correlation between the scoring functions of Gold, with the Gold flexible (**D**) and rigid (**E**) dockings and between G-score and G-score+Epik (**F**) with the Glide rigid docking. In these plots, for each compound, the score obtained by one method is reported *vs.* the score obtained with the other method. For Glide and Surflex, the scores are given as Δ*G* in kcal/mol, the lowest being the best, whereas for Gold, ChemPLP score and Goldscore are dimensionless fitnesses, with negative values for the former and positive values for the latter. Therefore, for the latter, the highest value is the best. The correlation coefficients between the methods are given in each plot.


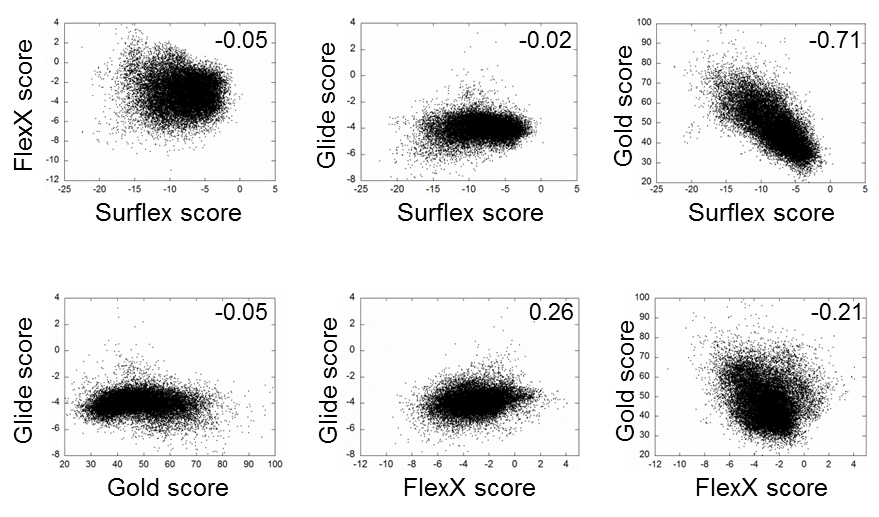


**Figure S2.** Same Figure legend as Figure 1 in the main text, but for Hbp instead of Cn.

**Table S1**: Correlation coefficients, *r*, between the rescoring results of Cn based on the poses obtained from Surflex, Glide, FlexX and Gold. For each set of poses, the scores were re-attributed by the four programs and then, the correlation between these scores was calculated for each pair of programs. For each set of poses, the highest correlation coefficient is written in bold type. Gold is expected to be anticorrelated (*r* < 0) with all the other programs because of the difference of signs in their scoring functions. However, this is not always the case. In addition, in some cases, Surflex and Glide are anticorrelated, although they should not be. This shows that with the rescoring, the correlations between the results were degraded.


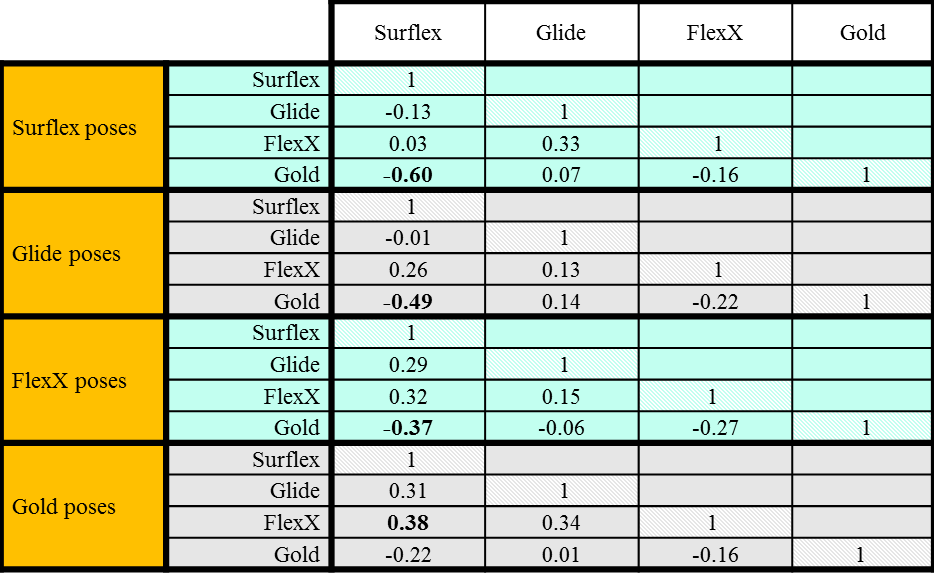


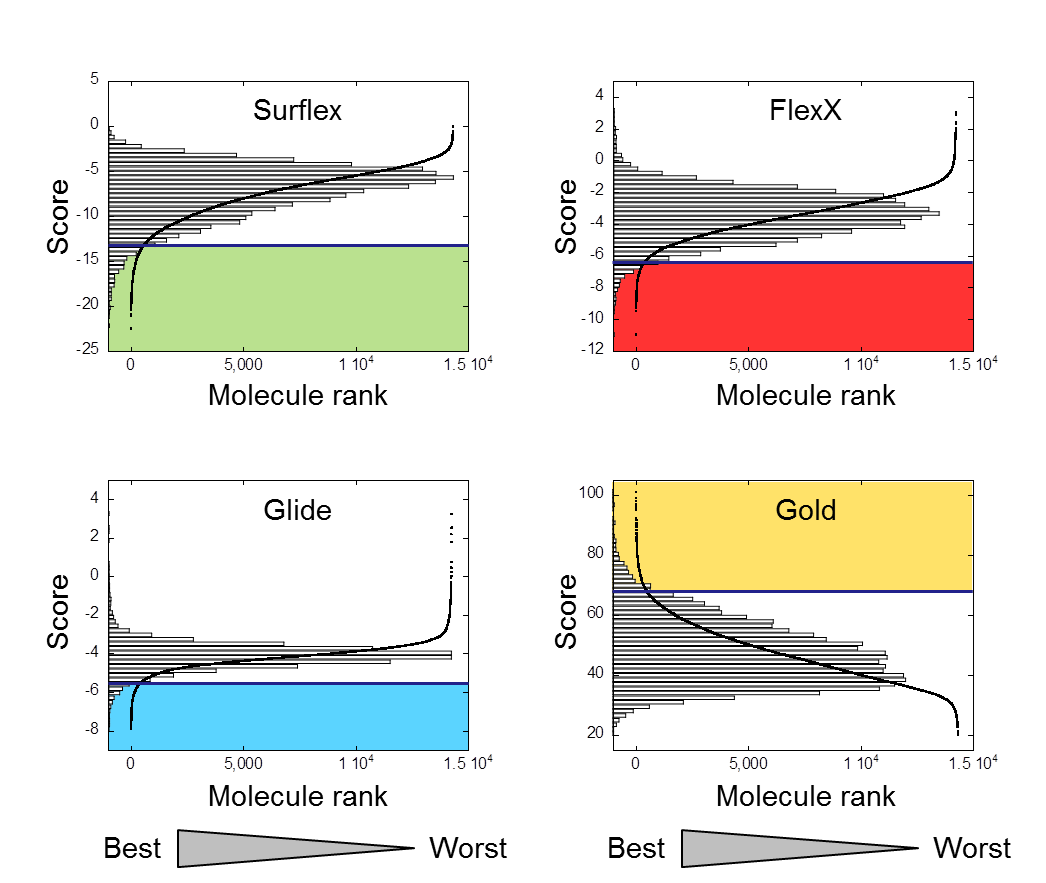


**Figure S3.** Same Figure legend as Figure 2 in the main text, but for Hbp instead of Cn.


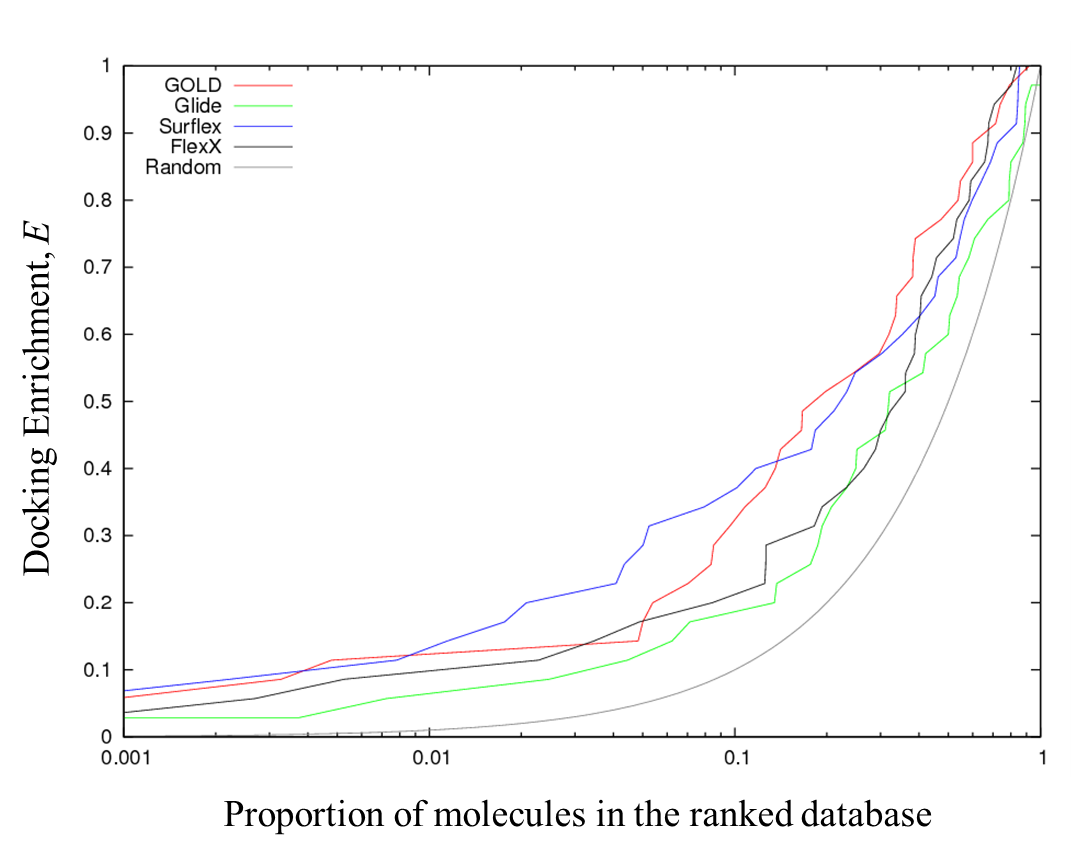


**Figure S4.** The docking enrichment, *E*, for cdk2 from Gold, Glide, Surflex and FlexX. *E* is defined as the proportion of hits found in the entire chemical library (see eq. 3 in the main text). It is drawn vs. the proportion of molecules tested in the ranked database. The scale is semi-logarithmic. In grey is reported the expected curve if the distribution of the hits was completely random. As observed in the corresponding rank curves (Figure 4 of the main text), the results obtained from the four programs are not completely random but close to it.


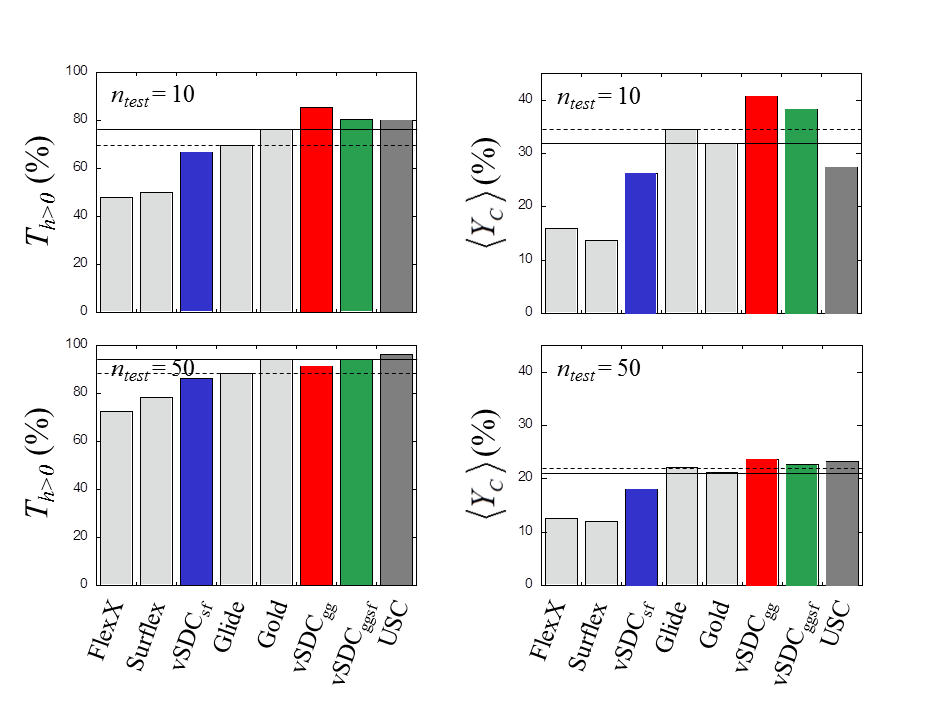


**Figure S5.** Summary of the results ($T_{h>0}$and $\left\langle Y_{C}\right\rangle$) for *n_test_* = 10 and *n_test_* = 50 for the DUD-E proteins. The horizontal lines delimit the values for Gold (plain line) and for Glide (dashed line). The bar colors are as follows: the isolated programs in light grey, vSDC_sf_, in blue, vSDC_gg_, in red, vSDC_ggsf_, in green and USC in dark grey. As observed, in all cases, {s; f} < vSDC_sf_ < {g; g}, whereas vSDC_gg_ > {g; g; s; f} except for $T_{h>0}$when *n_test_* = 50 (then vSDC_gg_ < {Gold}), and USC > {g; g; s; f}, except for $\left\langle Y_{C}\right\rangle$ when *n_test_* = 10 (then USC < {g; g}. However, vSDC_ggsf_ ≥ {g; g; s; f} in all cases.
